# PRISMA 2020 Is a Reporting Guideline for Systematic Reviews but Is Often Misused to Guide Review Content

**DOI:** 10.1002/cesm.70095

**Published:** 2026-08-09

**Authors:** Lasse Østengaard, Christoffer Bruun Korfitsen, Asbjørn Hróbjartsson, David Ruben Teindl Laursen, Camilla Hansen Nejstgaard

**Affiliations:** ^1^ Cochrane Denmark and Centre for Evidence‐Based Medicine Odense (CEBMO), Department of Clinical Research University of Southern Denmark Odense Denmark; ^2^ Open Patient data Explorative Network (OPEN) Odense University Hospital Odense Denmark

## Abstract

**Introduction:**

The Preferred Reporting Items for Systematic reviews and Meta‐Analyses (PRISMA) is a reporting guideline aiming to facilitate transparent and accurate reporting in systematic reviews. The latest version, PRISMA 2020, specifies that it is intended for guiding the reporting, not the conduct, of systematic reviews. We examined how often systematic review authors reported having used PRISMA 2020 outside its scope.

**Methods:**

We randomly sampled 200 systematic reviews citing PRISMA 2020 and extracted all sentences describing how PRISMA 2020 was used. Two authors independently assessed the extracted sentences and categorised the use of PRISMA 2020 as one of the following options: adequate (i.e., as a reporting guideline), inconclusive (e.g., unclear or ambiguous description) or inadequate (e.g., to guide review conduct).

**Results:**

The systematic reviews were published between 2021 and 2024, and 88.0% were published in biomedical journals. More than half (55.5%) of the systematic reviews described using PRISMA 2020 inadequately, typically as a guide for review conduct; 24.0% were categorised as ‘inconclusive’, and only 20.5% were categorised as ‘adequate’.

**Conclusion:**

More than half of the systematic reviews described using PRISMA 2020 outside its intended scope. We encourage systematic review authors to use PRISMA 2020 as a reporting guideline. Other resources are available to guide the conduct of systematic reviews, e.g., the Cochrane Handbook for Systematic Reviews of Interventions.

## 1 Introduction

Systematic reviews summarise and analyse empirical studies relevant to a specific research question. When adequately conducted and reported, they can be of great value to patients, clinicians, and decision‐makers. The Preferred Reporting Items for Systematic reviews and Meta‐Analyses (PRISMA) was published to facilitate transparent and accurate reporting in systematic reviews [1] and was later updated as PRISMA 2020 [2]. PRISMA 2020 is a reporting guideline, and this scope is clearly described in the core guideline paper, which includes: “*PRISMA 2020 is not intended to guide systematic review conduct…”*. [2].

Despite these clear instructions, we suspected that many researchers utilise the reporting guideline for purposes other than its intended use. This is problematic because researchers using PRISMA 2020 to guide systematic review conduct will not get adequate guidance on central aspects, such as literature searches and assessment of risk of bias in included studies.

Therefore, we decided to examine how often systematic review authors report having used PRISMA 2020 outside its scope as a reporting guideline.

## 2 Methods

We randomly sampled 200 systematic reviews citing PRISMA 2020 and extracted all sentences describing how PRISMA 2020 was used. Two authors independently assessed the extracted sentences and categorised the use of PRISMA 2020 as one of the following options (for further details, see Supplement A):

- ‘adequate’ (i.e., PRISMA 2020 was reported to be used as a reporting guideline)
- ‘inconclusive’ (e.g., the reported use of PRISMA 2020 was unclear or ambiguous)
- ‘inadequate’ (e.g., PRISMA 2020 was reported to be used to guide systematic review conduct)

We categorised the use of PRISMA 2020 as ‘inconclusive’ when extracted sentences could be categorised both as ‘adequate’ and ‘inadequate’ (this included, e.g., statements that the review was conducted and reported following PRISMA 2020), or when the categorisation was unclear (this included, e.g., reviews that solely stated to follow PRISMA 2020). Thus, a categorisation as ‘inconclusive’ did not imply that the reported use of PRISMA 2020 was inadequate.

## 3 Results

The 200 systematic reviews were published between 2021 and 2024, and most of them (88.0%) were published in biomedical journals. More than half (55.5%) of the systematic reviews described using PRISMA 2020 inadequately, 24.0% were categorised as ‘inconclusive’, and only 20.5% were categorised as ‘adequate’.

While systematic reviews categorised as ‘adequate’ clearly described that PRISMA 2020 was used to guide reporting, reviews categorised as ‘inadequate’ typically described using PRISMA 2020 to guide the conduct of the review (Table 1) [3, 4, 5, 6, 7, 8, 9, 10, 11, 12, 13, 14, 15].

**Table 1:** Examples of the use of PRISMA 2020.

*Examples*	*Quotes*
Example of adequate use of PRISMA 2020	*“The PRISMA 2020 statement was used as a guide for reporting this review [31].”* [3]
Examples of inconclusive use of PRISMA 2020	*“We followed the Preferred Reporting Items for Systematic Review and Meta‐Analysis (PRISMA) guidelines [12].”* [4]
	*“This review was conducted and reported following the Preferred Reporting Items for Systematic Reviews and Meta‐Analysis (PRISMA) guidelines [22]…”* [5]
Examples of inadequate use of PRISMA 2020	*“The design of this meta‐analysis followed the Preferred Reporting Items for Systematic reviews and Meta‐Analyses (PRISMA) guidelines. [24]”* [6]
	*“The Preferred Reporting Items for Systematic Reviews and Meta‐analyses (PRISMA) guidelines were used to guide the electronic search. [25]”* [7]
	*“Relevant studies were searched from PubMed and Web of Science databases in accordance with PRISMA guidelines …”* [8]
	*“According to Cochrane and PRISMA guidelines, two independent authors conducted data extraction.”* [9]
	*“The Preferred Reporting Items for Systematic Reviews and Meta‐Analyses (PRISMA) guidelines were strictly followed while conducting the meta‐analysis [19].”* [10]
	*“The database search, record screening, exclusion and eligibility guidelines were based on the approaches found in the Preferred Reporting Items for Systematic Reviews and Meta‐Analysis (PRISMA) [25].”* [11]
	*“The current review was performed following the requirements of the Preferred Reporting Items for Systematic Reviews and Meta‐Analysis (PRISMA) Statement [17].”* [12]
	*“PRISMA methodology for systematic reviews and meta‐analyses was followed …”* [13]
	*“This systematic review was conducted following the guidelines presented in the Preferred Reporting Items for Systematic Reviews and Meta‐Analyses (PRISMA) statement [40]…”* [14]
	*“Following PRISMA best‐practice guidelines, …”* [15]

Most of the systematic reviews categorised as inconclusive had an unclear description of how PRISMA 2020 was used. The authors used descriptions like ‘we followed the Preferred Reporting Items for Systematic Review and Meta‐Analysis (PRISMA) guidelines’ without further clarification. Other systematic reviews were categorised as inconclusive because PRISMA 2020 use was described ambiguously (Table 1).

## 4 Discussion

In our sample, more than half of the systematic reviews described using PRISMA 2020 for purposes other than its intended use, and only 20.5% reported using PRISMA 2020 as a reporting guideline.

PRISMA 2020 does not provide the methodological guidance needed when planning and conducting a systematic review. For example, PRISMA 2020 recommends describing the procedures for assessing risk of bias in the included studies [2], but it does not provide information on how to actually conduct the risk of bias assessment (e.g., related to which tools or criteria to use). If review authors assess risk of bias without having read and consulted the appropriate resources, they may do it incorrectly, which could lead to inadequate interpretation of a study's findings and thus misinform the readers. Also, PRISMA 2020 recommends presenting sources searched or consulted to identify studies [2], but it does not guide the selection of which sources to search. If systematic review authors only search in a few bibliographic databases (e.g., PubMed and Embase), even though searches in additional bibliographic databases and other sources would be appropriate for the research question, the review authors might miss relevant studies that potentially could change the conclusion of the review.

Our study's strengths are that we randomly sampled 200 systematic reviews citing PRISMA 2020, and two authors independently assessed and categorised the use of PRISMA 2020 based on predefined criteria. A limitation is the categorisation of the studies, which did not clearly indicate whether PRISMA 2020 was used adequately or inadequately. We categorised such reviews as ‘inconclusive’, acknowledging that they could have been categorised as ‘adequate’ when no clear signs of inadequate use were found.

Our findings may reflect a general misunderstanding that PRISMA 2020 implies preferred methods. Although intended as a reporting guideline, its recommendations of specific reporting items may be interpreted by authors as favouring certain methodological approaches. Additionally, other reporting guidelines may not have been as explicit about their scope as PRISMA 2020, and repeated descriptions of such guidelines as informing conduct may lead to it being adopted as the norm.

We suggest that our findings are a part of a larger problem concerning inadequate use of scientific references, which also includes selective use of references that support a specific hypothesis [16] and inappropriate self‐citations (e.g., excessive self‐citations to boost one's h‐index) [17]. Inadequate use of references is common in the biomedical literature [18], and using PRISMA 2020 to guide systematic review conduct falls into this category, as it misrepresents the original source.

Future research could investigate the extent to which inadequate use of PRISMA 2020 is reflected in the methodological quality of systematic reviews.

We encourage readers, peer reviewers, and editors to be aware of the risk that authors of systematic reviews cite and use PRISMA 2020 outside its intended scope. Researchers should use reporting guidelines like PRISMA 2020 to guide the reporting of their systematic reviews and use more appropriate resources like the Cochrane Handbook for Systematic Reviews of Interventions [19] and the JBI Manual for Evidence Synthesis [20] for methodological guidance.

## Author Contributions

**Lasse Østengaard:** conceptualization, methodology, writing – original draft, investigation, formal analysis, data curation, writing – review and editing. **Christoffer Bruun Korfitsen:** conceptualization, methodology, investigation, writing – review and editing, formal analysis. **Asbjørn Hróbjartsson:** conceptualization, methodology, writing – review and editing, formal analysis. **David Ruben Teindl Laursen:** conceptualization, formal analysis, writing – review and editing, methodology, investigation. **Camilla Hansen Nejstgaard:** conceptualization, methodology, investigation, formal analysis, writing – review and editing.

## Funding

The authors have nothing to report.

## Conflicts of Interest

The authors declare no conflicts of interest.

## Supporting information


Supporting File

## Data Availability

The data that support the findings of this study are available from the corresponding author upon reasonable request.

## References

[1] D. Moher , A. Liberati , J. Tetzlaff , et al., “Preferred Reporting Items for Systematic Reviews and Meta‐Analyses: The PRISMA Statement,” Annals of Internal Medicine 151, no. 4 (2009): 264–269, 10.7326/0003-4819-151-4-200908180-00135. 19622511

[2] M. J. Page , J. E. McKenzie , P. M. Bossuyt , et al., “The PRISMA 2020 Statement: An Updated Guideline for Reporting Systematic Reviews,” BMJ 372 (2021): n71, 10.1136/bmj.n71. 33782057 PMC8005924

[3] C. Sherlock , C. Madigan , C. Linehan , et al., “Academic Attainment Following Pediatric Epilepsy Surgery: A Systematic Review,” Epilepsy & Behavior 134 (2022): 108847, 10.1016/j.yebeh.2022.108847. 35914437

[4] Y. Liu , W. Li , Y. Chen , et al., “Anti‐CD3 Monoclonal Antibodies in Treatment of Type 1 Diabetes: A Systematic Review and Meta‐Analysis,” Endocrine 2024 83, no. 2: 322–329, 10.1007/s12020-023-03499-0. 37658243

[5] S. Kiconco , C. T. Tay , K. L. Rassie , et al., “Natural History of Polycystic Ovary Syndrome: A Systematic Review of Cardiometabolic Outcomes From Longitudinal Cohort Studies,” Clinical Endocrinology (Oxford) 96, no. 4 (2022): 475–498, 10.1111/cen.14647. 34894357

[6] Y. Jiang , Z. Chen , Y. Chen , et al., “Low‐Dose Asprin Use During Pregnancy May Be a Potential Risk for Postpartum Hemorrhage and Increased Blood Loss: A Systematic Review and Meta‐Analysis,” American Journal of Obstetrics & Gynecology MFM 5, no. 4 (2023): 100878, 10.1016/j.ajogmf.2023.100878. 36706919

[7] D. M. Malinga , A. E. Laher , J. McDowall , et al., “Coronavirus Disease 2019 (COVID‐19) and Priapism: An Unexplored Association,” Current Urology 16, no. 2 (2022): 55–62, 10.1097/CU9.0000000000000111. 35789564 PMC9245531

[8] R. Timon , I. Martinez‐Guardado , and F. Brocherie , “Effects of Intermittent Normobaric Hypoxia on Health‐Related Outcomes in Healthy Older Adults: A Systematic Review,” Sports Medicine ‐ Open 9, no. 1 (2023): 19, 10.1186/s40798-023-00560-0. 36843041 PMC9968673

[9] A. Aiyar , T. K. Pedersen , C. M. Resnick , et al., “Management of Unilateral Craniofacial Microsomia With Orthopaedic Functional Appliances: A Systematic Literature Review,” Orthodontics & Craniofacial Research 27, no. Suppl 1 (2024): 131–140, 10.1111/ocr.12729. 37987216

[10] L. Zhang , C. Chen , D. Chai , et al., “The Association Between Antibiotic Use and Outcomes of HCC Patients Treated With Immune Checkpoint Inhibitors,” Frontiers in Immunology 13 (2022): 956533, 10.3389/fimmu.2022.956533. 36059512 PMC9429218

[11] D. DeArmond , F. Emmert , A. C. M. Pinto , et al., “A Systematic Review of Logging Impacts in the Amazon Biome,” Forests 14, no. 1 (2023): 81, 10.3390/f14010081.

[12] M. Radic , E. Kolak , H. Dogas , et al., “Body Composition Parameters in Systemic Sclerosis ‐ a Systematic Review and Meta‐Analysis,” Rheumatology (Oxford, England) 63, no. 1 (2024): 16–25, 10.1093/rheumatology/kead418. 37647631

[13] W. Al Arashi , L. G. R. Romano , F. W. G. Leebeek , et al., “Desmopressin to Prevent and Treat Bleeding in Pregnant Women With an Inherited Bleeding Disorder: A Systematic Literature Review,” Journal of Thrombosis and Haemostasis 22, no. 1 (2024): 126–139, 10.1016/j.jtha.2023.09.021. 37778511

[14] T. I. M. Aarsland , J. T. Instanes , M. R. Posserud , et al., “Changes in Tryptophan‐Kynurenine Metabolism in Patients With Depression Undergoing ECT ‐ A Systematic Review,” Pharmaceuticals (Basel) 15, no. 11 (2022): 1439, 10.3390/ph15111439. 36422569 PMC9694349

[15] R. Wincza , C. Hartley , J. Fenton‐Romdhani , et al., “The Development of Susceptibility to Geometric Visual Illusions in Children – A Systematic Review,” Cognitive Development 69 (2024): 101410, 10.1016/j.cogdev.2023.101410.

[16] S. A. Greenberg , “How Citation Distortions Create Unfounded Authority: Analysis of a Citation Network,” BMJ 339 (2009): b2680, 10.1136/bmj.b2680. 19622839 PMC2714656

[17] J. P. Ioannidis , “A Generalized View of Self‐Citation: Direct, Co‐Author, Collaborative, and Coercive Induced Self‐Citation,” Journal of Psychosomatic Research 78, no. 1 (2015): 7–11, 10.1016/j.jpsychores.2014.11.008. 25466321

[18] C. Baethge and H. Jergas , “Systematic Review and Meta‐Analysis of Quotation Inaccuracy in Medicine,” Research Integrity and Peer Review 10, no. 1 (2025): 13, 10.1186/s41073-025-00173-z. 40696441 PMC12285159

[19] J. P. T. Higgins , J. Thomas , J. Chandler , and V. A. Welch , ed., Cochrane Handbook for Systematic Reviews of Interventions version 6.4 (updated August 2023). Cochrane, 2023. Available from www.training.cochrane.org/handbook.

[20] E. Aromataris , C. Lockwood , K. Porritt , B. Pilla , and Z. Jordan , ed., JBI Manual for Evidence Synthesis. JBI, 2024. Available from: https://synthesismanual.jbi.global.

