# Supplementary material for: PRISMA 2020 Is a Reporting Guideline for Systematic Reviews but Is Often Misused to Guide Review Content
Source: Cochrane Evid Synth Methods. 2026 Aug 9;4(5):e70095. doi: 10.1002/cesm.70095 (PMC13459888; doi:10.1002/cesm.70095)
Supplement: Supplementary file 1 [file CESM-4-e70095-s001.docx]

**Supplementary materials for the manuscript entitled:** PRISMA 2020 is a reporting guideline for systematic reviews but is often misused to guide review content

Table of contents

[Supplement A - Methodology	2](#_Toc207117200)

[Reference list	4](#_Toc207117201)

## Supplement A - Methodology

*Study design*

A cross-sectional study of systematic reviews citing PRISMA 2020.

*Eligibility criteria*

We included publications defined as a “systematic review” by the review authors (for example, in the title or methods section) and with an explicit reference to PRISMA 2020.^1^ We included systematic reviews published after March 2021, as PRISMA 2020 was published on March 29, 2021.

We excluded systematic reviews written in languages other than English and those in which PRISMA 2020 was only cited or mentioned in relation to a figure or table.

*Identifying the systematic reviews*

The PRISMA 2020 statement was published simultaneously in the following five journals^[1]^:

- Journal of Clinical Epidemiology
- British Medical Journal
- International Journal of Surgery
- PLOS Medicine
- Systematic Reviews

In Web of Science, we exported five reference lists (one for each journal) of articles that have cited the PRISMA 2020 statement publication.^1-5^ We conducted the search in Web of Science on March 25, 2024, and retrieved 26,183 references.

*Management and screening of the systematic reviews*

All references were imported into Covidence, where they received an ID number.

We then used a random sequence generator (https://www.randomdraws.com/random-sequence-generator/) to generate a random sample. A single person (LØ) screened the references for inclusion, and the procedure was repeated until we had identified 200 eligible systematic reviews.

The excluded references were tagged with:

- Wrong language
- Not a systematic review
- PRISMA Extension
- Protocol for a systematic review

*Data extraction*

A single person (LØ) extracted all sentences describing how PRISMA 2020 was used in the 200 systematic reviews.

We extracted sentences in which the authors explicitly stated “PRISMA” (with and without 2020), “Preferred Reporting Items for Systematic reviews and Meta-Analyses”, or inserted an in-text citation referring to the PRISMA 2020 statement publication. The sentences were extracted into an Excel sheet together with meta-data on the systematic review’s ID number, journal name, research field (health research or non-health research), and publication year.

Two persons (LØ and either CHN, CBK, or DRTL) independently categorised each sentence as:

| **Adequate** | **Inconclusive** | **Inadequate** |
| --- | --- | --- |
| - PRISMA 2020 was described to be used to guide the reporting of the systematic review. | - There was no clear description of how PRISMA 2020 was used. | - PRISMA 2020 was described to be used to guide the conduct of the systematic review. - PRISMA 2020 was described to be used as a tool to assess the conduct or methodological quality. - Other inadequate ways. |

The sentences were categorised as “adequate” if the authors of the systematic review had described using PRISMA 2020 as a reporting guideline.

The sentences were categorised as “inconclusive” if the authors of the systematic review had made an unclear description of how PRISMA 2020 was used (e.g., “we followed the PRISMA statement in our systematic review”).

The sentences were categorised as “inadequate” if the authors of the systematic review had described the use of PRISMA 2020 for purposes other than what PRISMA 2020 is intended for.

Moreover, two persons (LØ and either CHN, CBK, or DRTL) independently categorised each sentence as “general” (e.g., *we reported our systematic review in accordance with the PRISMA 2020 statement*), “item specific” (e.g., *following the PRISMA 2020 statement we conducted our search…*), or “generic” (e.g., table/figure legends). Sentences categorised as “generic” were not included in the overall categorisation of the systematic review.

If a systematic review contained multiple sentences or descriptions of PRISMA 2020 use, the systematic review was categorised based on the following rules:

***Adequate:***

a) All the extracted sentences were categorised as adequate.
b) The extracted sentences were categorised as adequate and inclusive.

***Inconclusive:***

a) All the extracted sentences were categorised as inconclusive.
b) The extracted sentences were categorised as both adequate and inadequate (with an even distribution).

***Inadequate:***

a) All the extracted sentences were categorised as inadequate.
b) The extracted sentences were categorised as inadequate and inconclusive.

Disagreements between the two persons were solved by discussion.

Our data extraction sheet was pilot-tested (by LØ and CHN), using 10 randomly selected systematic reviews (not included in our sample) before we started extracting references from the Web of Science.

*Data synthesis*

We estimated the number and proportion of the systematic reviews categorised as adequate, inconclusive, and inadequate. For systematic reviews categorised inconclusive, we provided a qualitative description of the main reasons for the categorisation. Furthermore, we provided illustrative quotes for systematic reviews that were categorised as adequate, inconclusive, and inadequate.

### Reference list

1. Page MJ, McKenzie JE, Bossuyt PM, et al. The PRISMA 2020 statement: an updated guideline for reporting systematic reviews. BMJ 2021;372:n71. doi: 10.1136/bmj.n71.

2. Page MJ, McKenzie JE, Bossuyt PM, et al. The PRISMA 2020 statement: An updated guideline for reporting systematic reviews. PLoS Med. 2021;18(3):e1003583. doi: 10.1371/journal.pmed.1003583

3. Page MJ, McKenzie JE, Bossuyt PM, et al. The PRISMA 2020 statement: an updated guideline for reporting systematic reviews. Syst Rev. 2021;10(1):89. doi: 10.1186/s13643-021-01626-4

4. Page MJ, McKenzie JE, Bossuyt PM, et al. The PRISMA 2020 statement: An updated guideline for reporting systematic reviews. J Clin Epidemiol. 2021;134:178-89. doi: 10.1016/j.jclinepi.2021.03.001

5. Page MJ, McKenzie JE, Bossuyt PM, et al. The PRISMA 2020 statement: An updated guideline for reporting systematic reviews. Int J Surg. 2021;88:105906. doi: 10.1016/j.ijsu.2021.105906

6. Page MJ, McKenzie JE, Bossuyt PM, et al. The PRISMA 2020 statement: an updated guideline for reporting systematic reviews. Rev Esp Cardiol (Engl Ed). 2021;74(9):790-9. doi: 10.1016/j.rec.2021.07.010

1. We did not retrieve a reference list from the journal Revista Española de Cardiología in which the Spanish version of PRISMA 2020 was published.^6^ Page MJ, McKenzie JE, Bossuyt PM, et al. The PRISMA 2020 statement: an updated guideline for reporting systematic reviews. *Rev Esp Cardiol (Engl Ed)* 2021;74(9):790-99. doi: 10.1016/j.rec.2021.07.010
